# Supplementary material for: Identification of 37 Heterogeneous Drug Candidates for Treatment of COVID-19 via a Rational Transcriptomics-Based Drug Repurposing Approach
Source: Pharmaceuticals (Basel). 2021 Jan 25;14(2):87. doi: 10.3390/ph14020087 (PMC7912585; doi:10.3390/ph14020087)
Supplement: Supplementary file 1 [file pharmaceuticals-14-00087-s001.zip › COVID19_Supplementary_Files_pharmaceuticals-1083626_revision1/COVID19_Supplementary_Figures_pharmaceuticals-1083626_revision1.pdf]

Supplementary Figures

Figure S1. Selection of the relevant datasets (detailed pipeline)

Figure S2. Minor portion of DEGs is shared among multiple datasets. While most of the DEGs are unique to a single dataset (N = 1 on x-axes), a decreasing number of shared DEGs is identified as the number of overlapped datasets increases. (a) Number of shared DEGs obtained from each dataset (**Table S1**); (b) N of shared DEGs obtained from each group of datasets (three cell lines with two different MOI and for a combination of NHBE cells and hBO, **Table S2**)

Figure S3. The PCA score plots for the three cell lines with two different MOIs and for a combination of NHBE cells and hBO. (a) A549-ACE2; (b) Calu-3; (c) A549; (d) NHBE and hBO

Figure S4. Hierarchical clustering of various biosamples based on transcriptomic signature changes upon SARS-CoV-2 infection

Figure S5. Selection of the relevant DEGs (detailed pipeline)

Figure S6. Final list of consensus DEGs upon SARS-CoV-2 infection

Figure S7. Selection of the drugs (detailed pipeline)

Figure S8. Distribution of 37 repurposable drug candidates with a potential to reverse transcriptomic signature upon SARS-CoV-2 infection based on their properties. (a) Current pharmacological class and therapeutic indication; (b) Current mechanism of action (MOA)

Figure S9. Hierarchical clustering of 37 drug candidates based on molecular structure

Figure S10. PCA biplot demonstrating heterogeneity of 37 drugs in physicochemical space. Drugs are coloured according to an estimated ionised state at pH 7.4

Figure S11. Distribution of 37 drug candidates based on drug target properties. (a) Protein superfamilies of drug target; (b) Cellular location of drug target

Figure S12. Hierarchical clustering of 37 drug candidates based on combined properties (pharmacological class and current indication, mechanism of action (MOA), cellular location, protein superfamilies of drug targets, and biological pathways in overlap between pathways regulated by 37 drug candidates and pathways affected by SARS-CoV-2 virus)

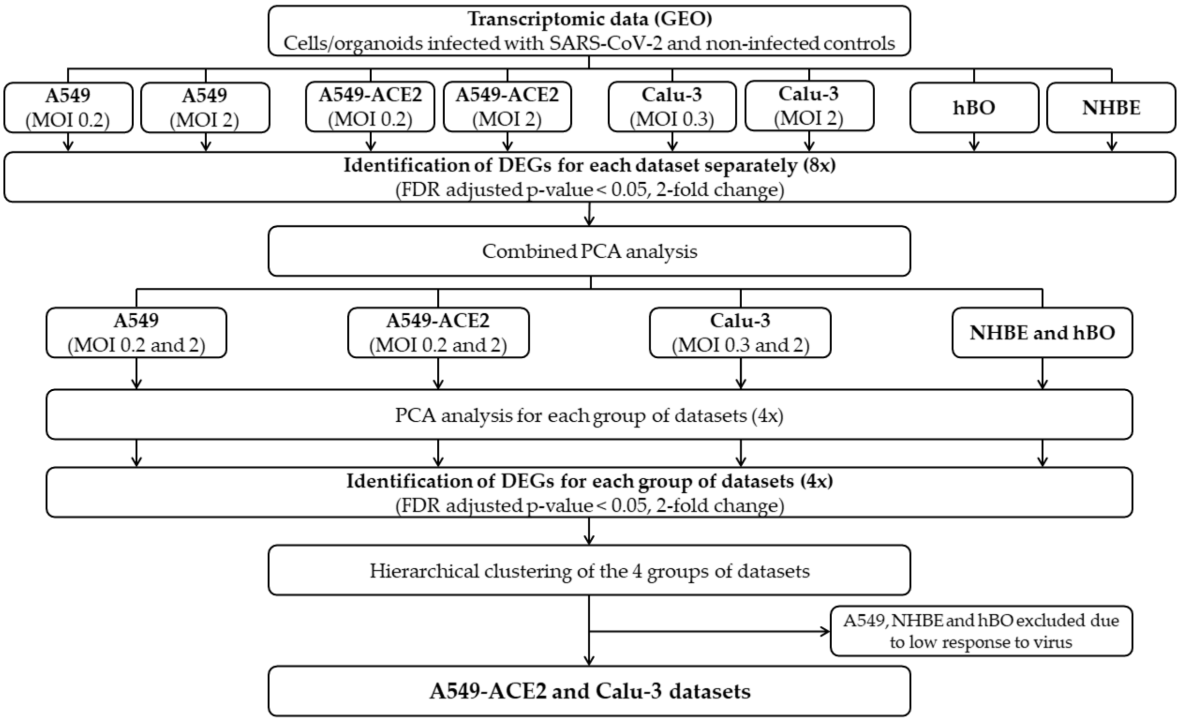

Figure S1. Selection of the relevant datasets (detailed pipeline)

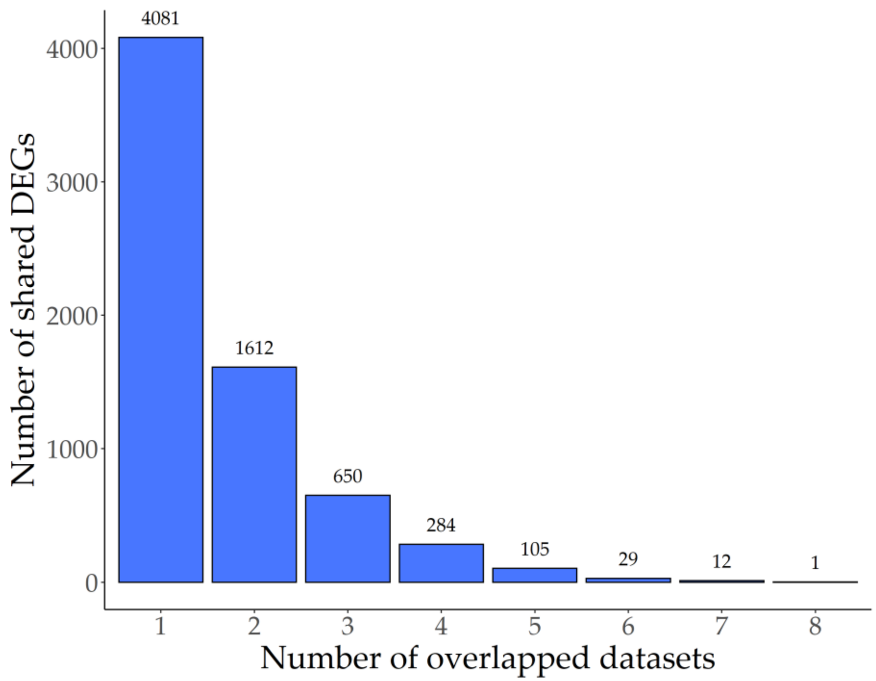

(a)

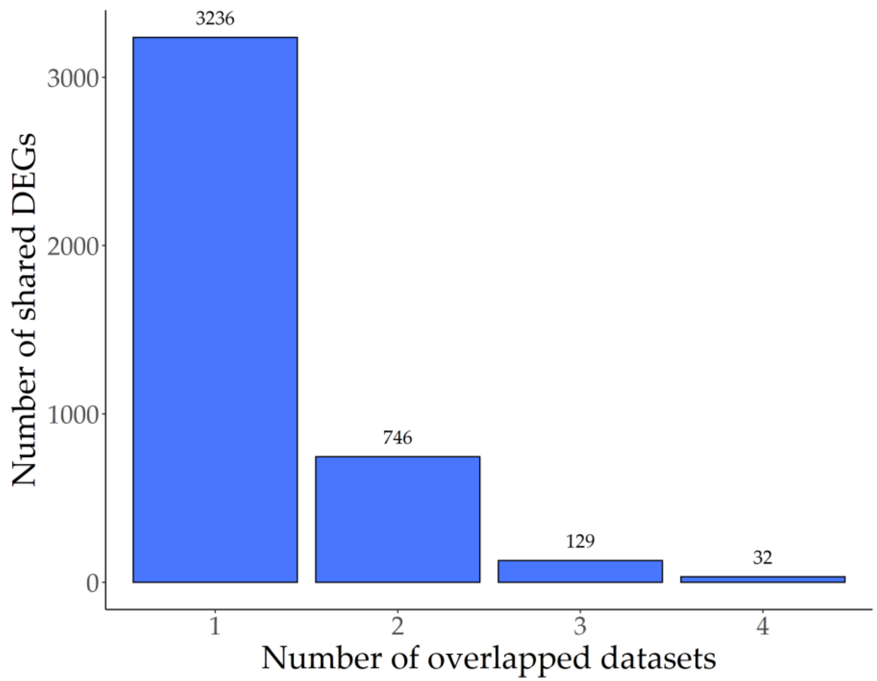

(b)

Figure S2. Minor portion of DEGs is shared among multiple datasets. While most of the DEGs are unique to a single dataset (N = 1 on x-axes), a decreasing number of shared DEGs is identified as the number of overlapped datasets increases. **(a)** Number of shared DEGs obtained from each dataset (**Table S1**); **(b)** N of shared DEGs obtained from each group of datasets (three cell lines with two different MOI and for a combination of NHBE cells and hBO, **Table S2**).

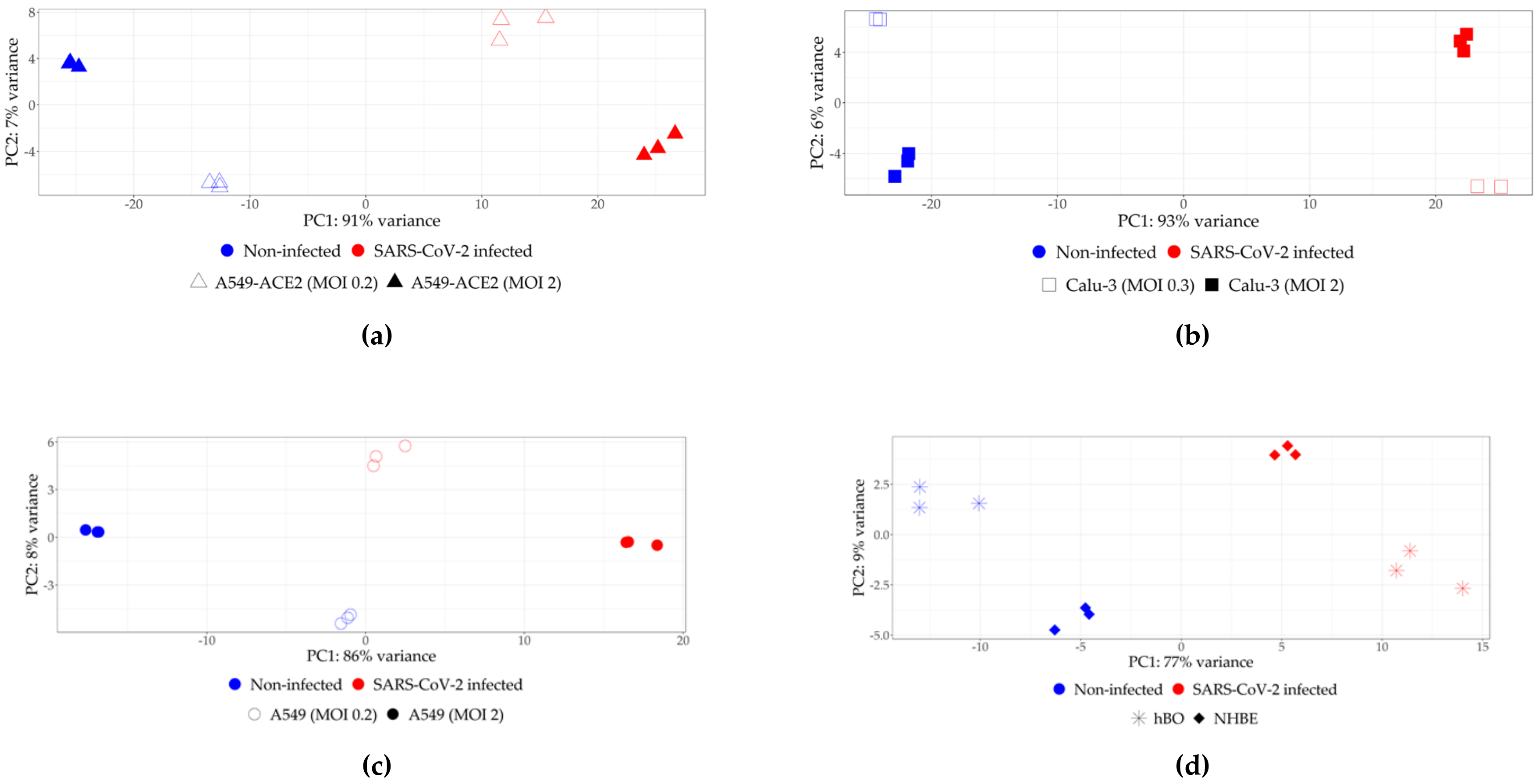

Figure S3. The PCA score plots for the three cell lines with two different MOIs and for a combination of NHBE cells and hBO. **(a)** A549-ACE2; **(b)** Calu-3; **(c)** A549; **(d)** NHBE and hBO

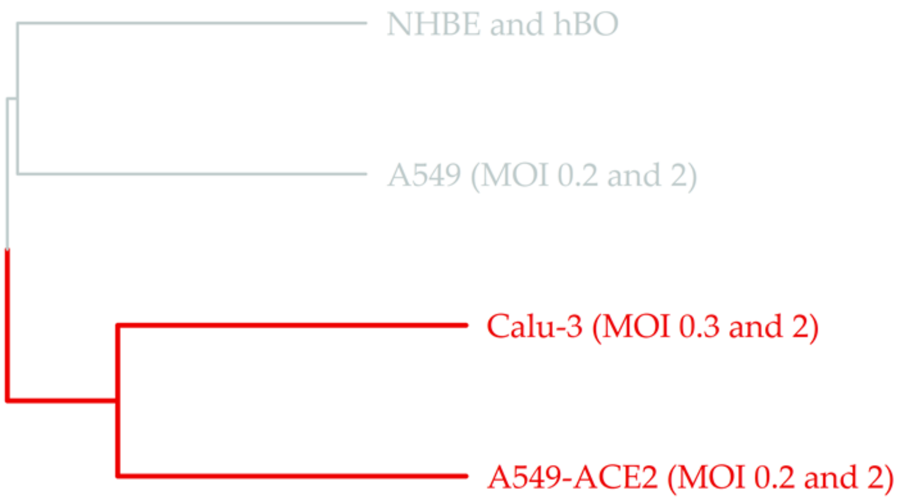

Figure S4. Hierarchical clustering of various biosamples based on transcriptomic signature changes upon SARS-CoV-2 infection

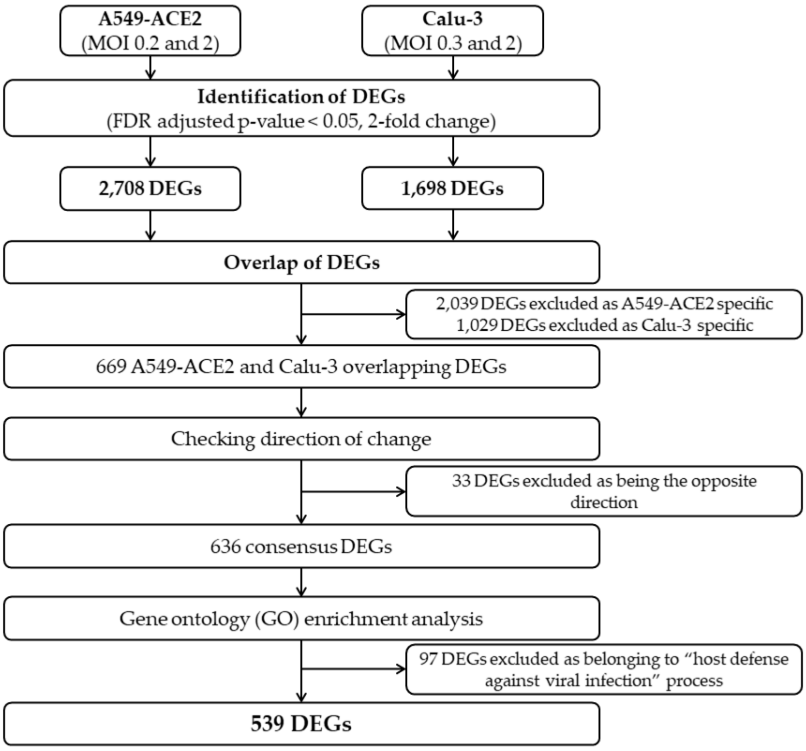

Figure S5. Selection of the relevant DEGs (detailed pipeline)

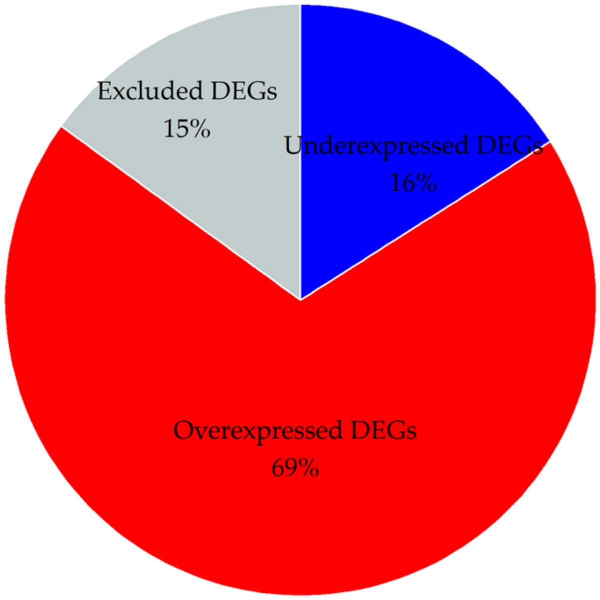

Figure S6. Final list of consensus DEGs upon SARS-CoV-2 infection

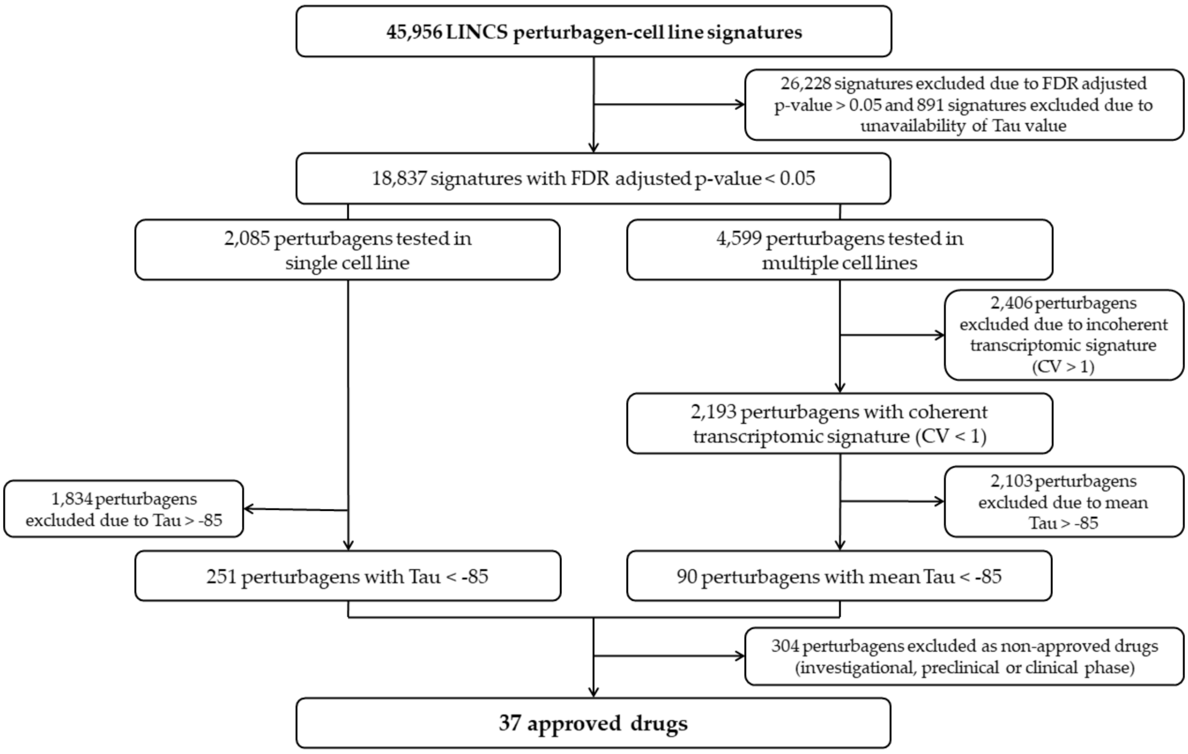

Figure S7. Selection of the drugs (detailed pipeline)

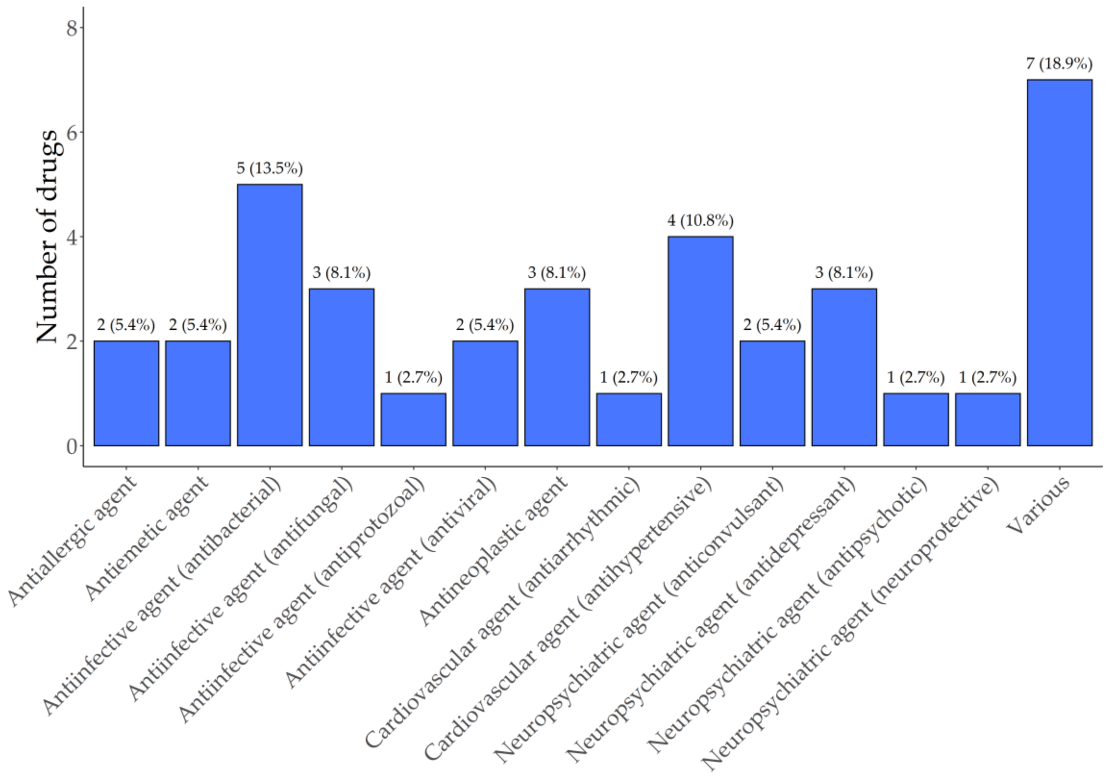

(a)

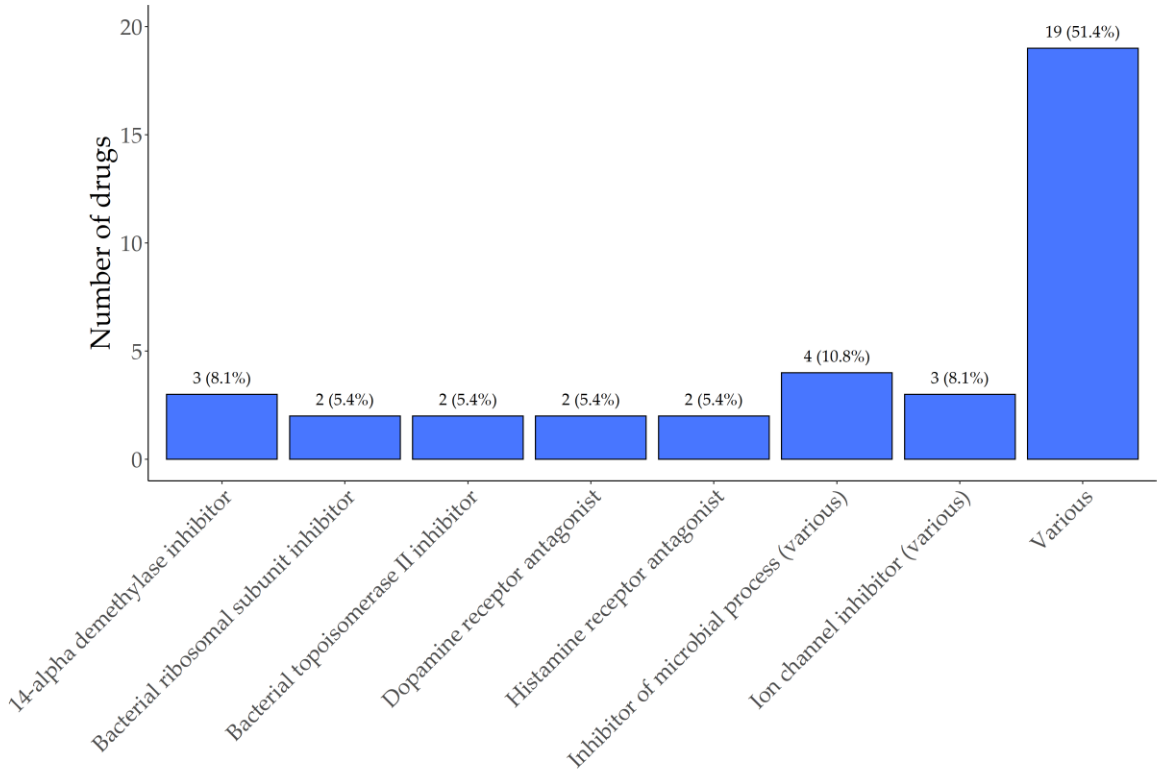

(b)

Figure S8. Distribution of 37 repurposable drug candidates with a potential to reverse transcriptomic signature upon SARS-CoV-2 infection based on their properties. **(a)** Current pharmacological class and therapeutic indication; **(b)** Current mechanism of action (MOA)

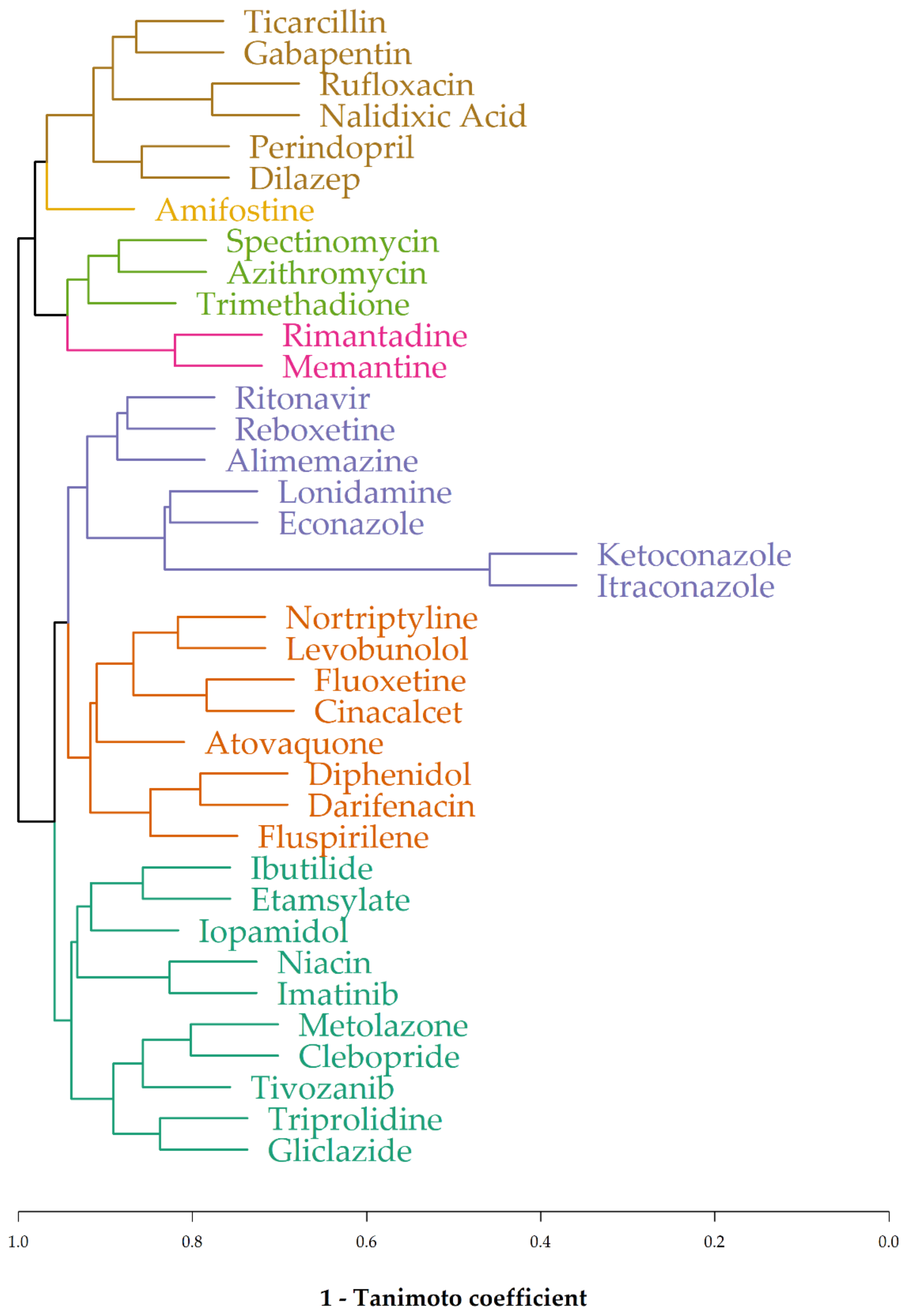

Figure S9. Hierarchical clustering of 37 drug candidates based on molecular structure

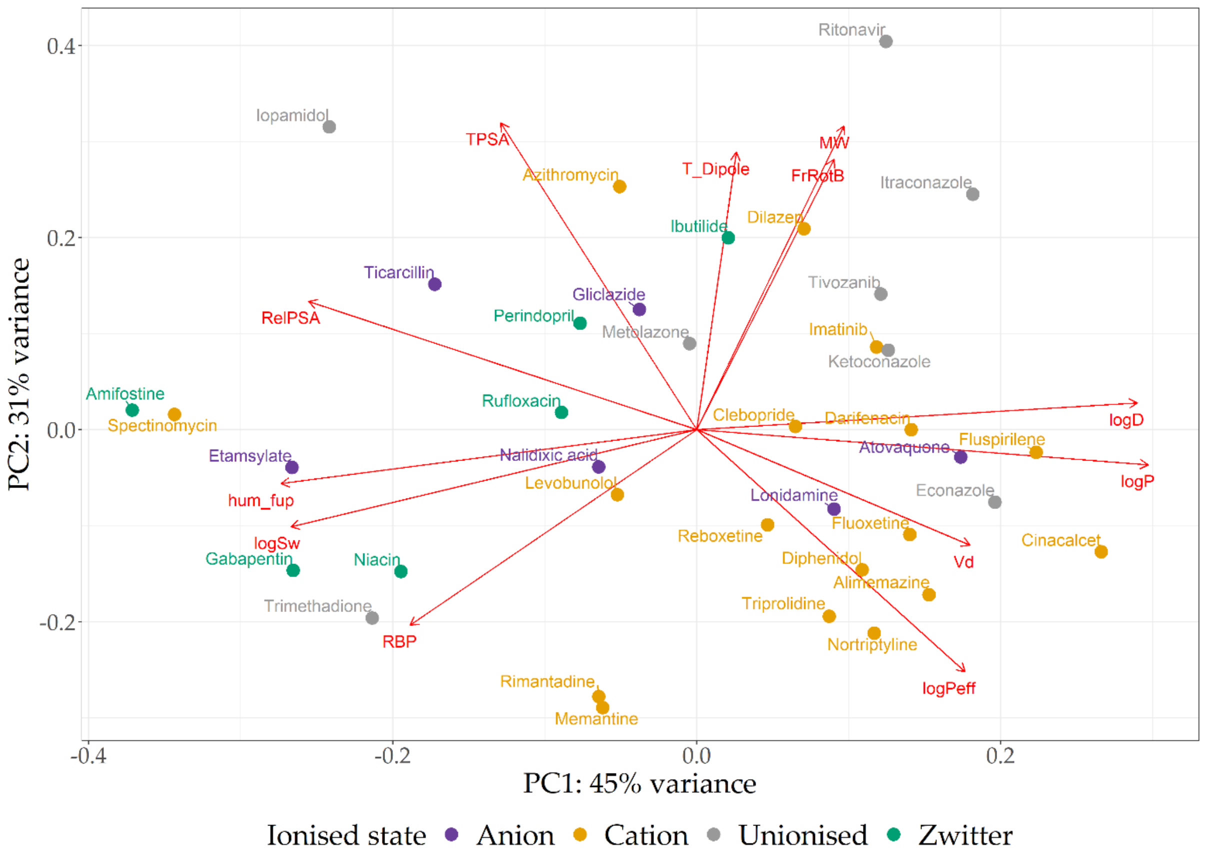

Figure S10. PCA biplot demonstrating heterogeneity of 37 drugs in physicochemical space. Drugs are coloured according to an estimated ionised state at pH 7.4

Abbreviations: MW – molecular weight; logP – logarithm of n-octanol/water lipophilicity coefficent; logD – logarithm of distribution coefficient for pH=7.4; FrRotB – a number of free rotable bonds; logPeff – logarithm of passive mebrane permeability (cm/s * 10^{-4}); logSw – logarithm of native water solubility (mol/L); T_Dipole – topological dipole moment; TPSA – topological polar surface area; RelPSA – relative polar surface area; hum_fup – fraction of free drug in human plasma (%); RBP – blood-to-plasma ratio in human; Vd – volume of distribution at the steady state (L/kg).

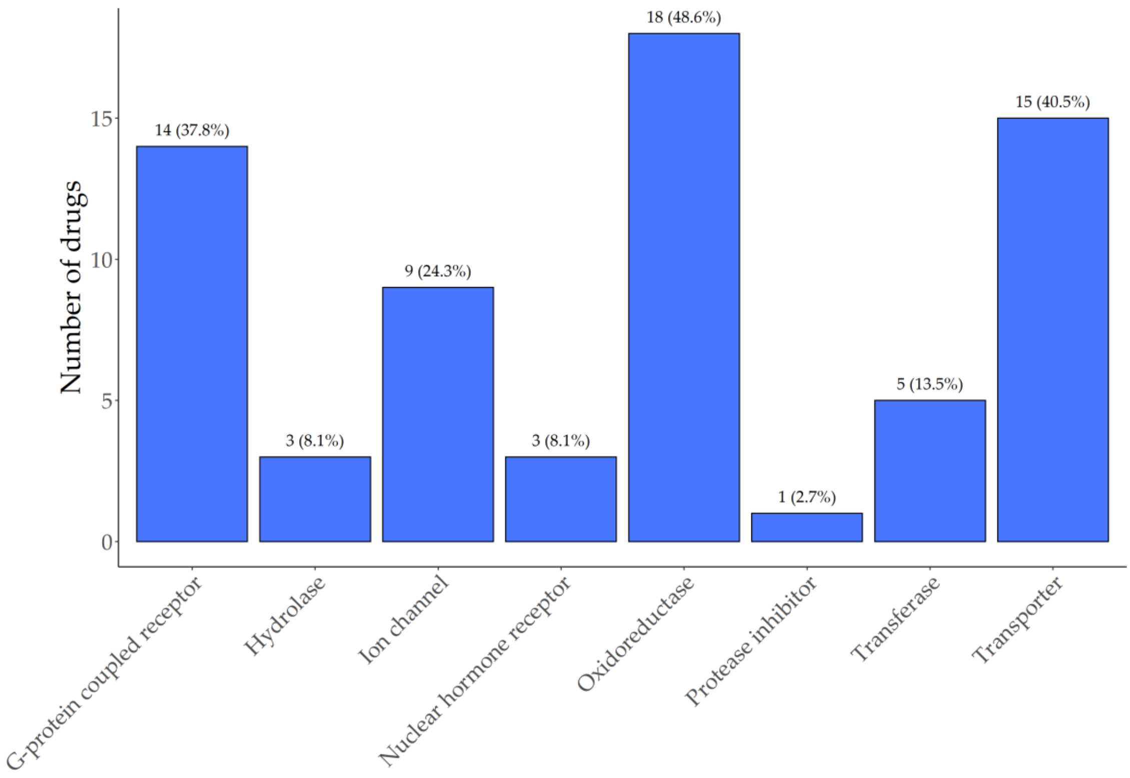

(a)

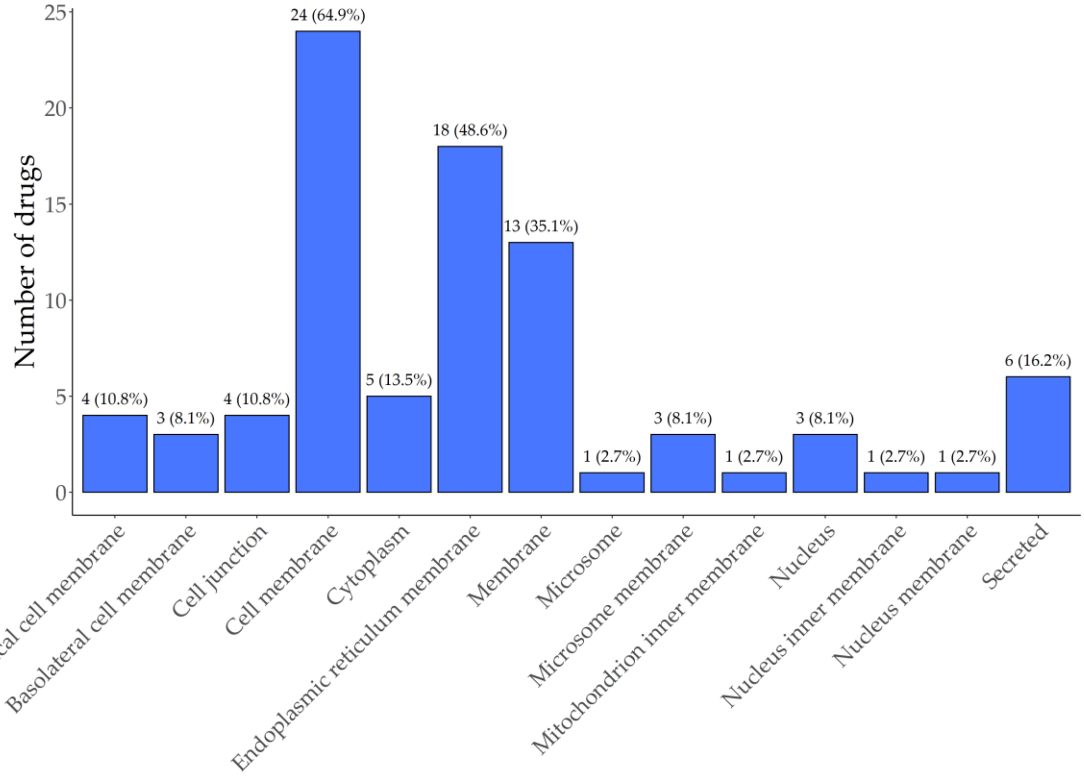

(b)

Figure S11. Distribution of 37 drug candidates based on drug target properties. **(a)** Protein superfamilies of drug target; **(b)** Cellular location of drug target

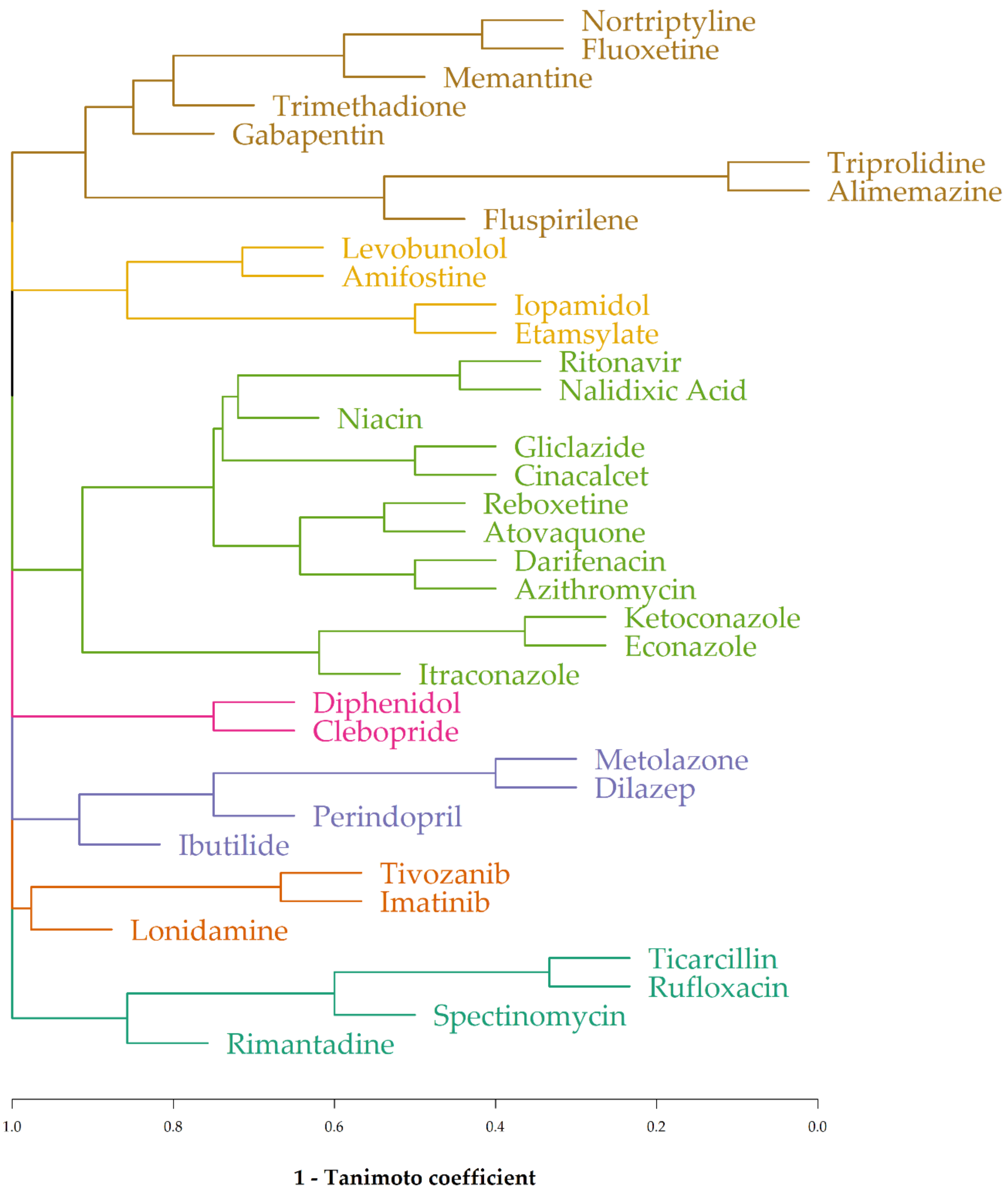

Figure S12. Hierarchical clustering of 37 drug candidates based on combined properties (pharmacological class and current indication, mechanism of action (MOA), cellular location, protein superfamilies of drug targets, and biological pathways in overlap between pathways regulated by 37 drug candidates and pathways affected by SARS-CoV-2 virus)
